# Supplementary material for: Chinese Pedigree with Hereditary Gastrointestinal Stromal Tumors: A Case Report and Literature Review
Source: Int J Mol Sci. 2023 Jan 3;24(1):830. doi: 10.3390/ijms24010830 (PMC9820900; doi:10.3390/ijms24010830)

Figure S1: Negative immunohistochemistry results of the proband's largest lesion.

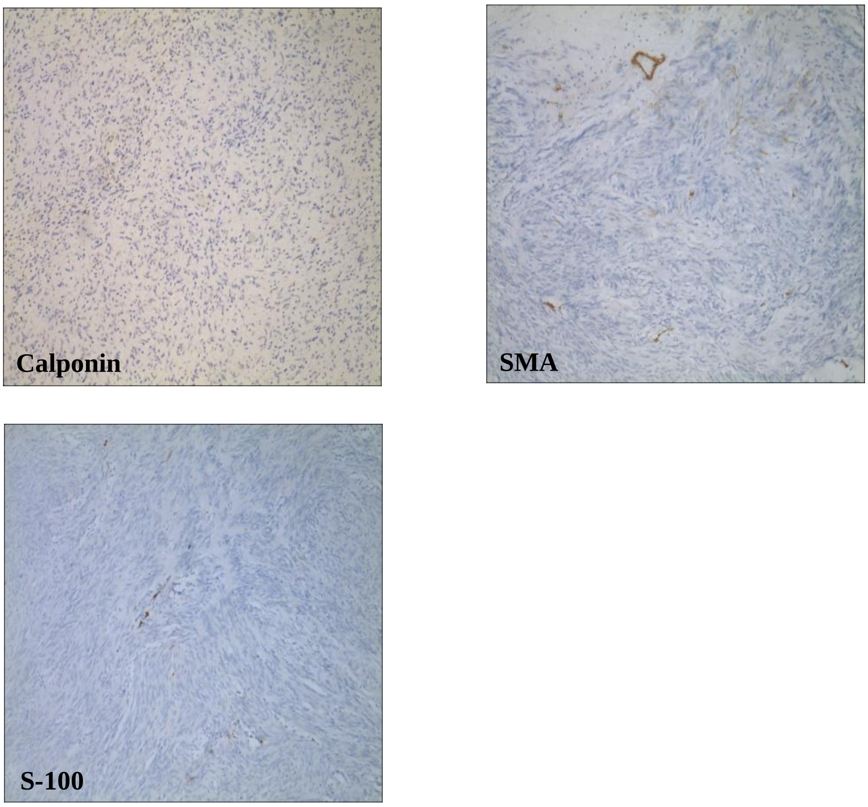

Figure S2: Gross specimen of the total gastrectomy.

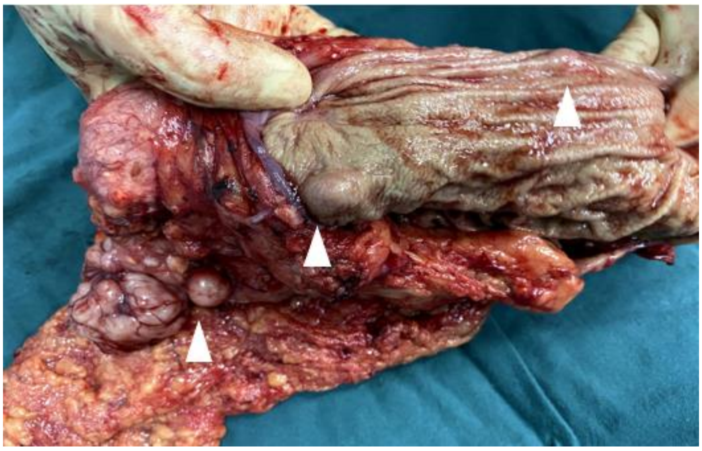

Figure S3: Dose-finding experiments of HEK 293T cell line. For cell viability assays, 3000 cells were plated in 96-well plates and cultured overnight. Compounds (imatinib, ripretinib, avapritinib) were then added in serial dilutions. Cellular ATP levels

were determined after 48h by the Cell Titer-Glo® Luminescent Cell Viability Assay (cat# G7570, Promega, Madison, WI, USA). The absorbance of the plates was measured on a THERMO max microplate reader.

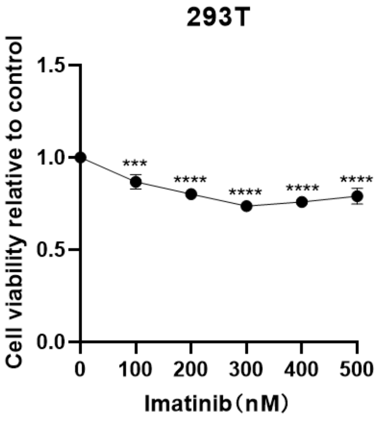

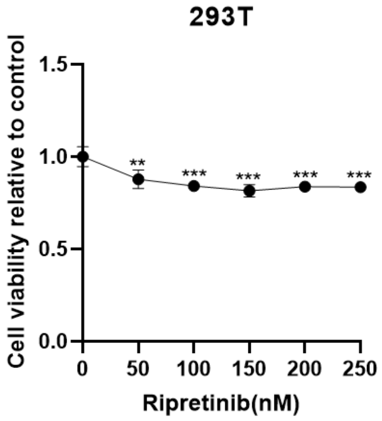

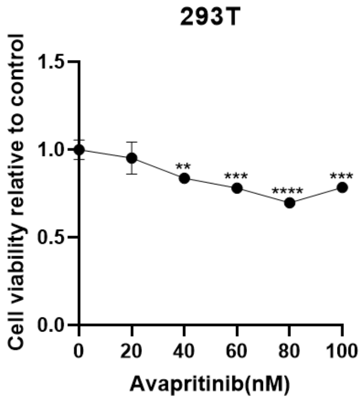

Supplement: Supplementary file 1 [file ijms-24-00830-s001.zip › ijms-2090840-supplementary.pdf]
